# A Trip on the Railroad

**Published:** 1851-10

**Authors:** 


### Editorial.

A Trip on the Railroad.—We had the pleasure of a short ride, as far as Columbus, on the railroad, and were much gratified to find the Profession in a flourishing condition. Dr. Ide, of Columbus, was busily engaged on a set of teeth, with patients waiting to have teeth plugged and extracted. Drs. Riley and Satterthwait were both out of the City, and hence we availed ourself of Dr. Ide's kindness in the offer of his horse and buggy to visit the Blind Asylum, Penitentiary and Lunatic Asylum. At the former it was vacation, and hence not much of interest could be seen except a few specimens of their handywork, which would appear to require not only good sight but good fingers. At the two latter places they never have vacation. We noticed some of the inmates of the Penitentiary have bad teeth. But we would suppose the diet given them in prison never induced the disease. This consists of coarse bread and fat meat, with, we believe, a dessert of rice; fare nutritious enough, but not such as would be selected by epicures. The State has a large workshop, and has rented it out as we think very cheap; one gentlemen having the most of it under his control—the State furnishing him two hundred hands at forty cents per day—charging nothing for board and shop rent. Some appear to be expert workmen, and might anywhere make $1 to $2 per day. We judge it to be a poor place for a dentist; yet, we believe, they keep a *doctor* and *barber*. A solitary, silent and gloomy abode—pent up vice—the effervescence of crime imprisoned and made to succumb to law. Could we trace out each history, how much of the misery we see might be ascribed to defective parental education.

The Lunatic Asylum—we hastily passed through the male apartments—nor did we feel much disposed to linger in any of the apartments—wrecked intellect—how strange and how diversant the cause of reason's dethronement—yet she may be pulled from the throne, by ambition defeated, by imagination's wild revelries unrestrained—by passion debased—truly a pitable sight. Yet all this congregated perverted intellect—this morose, sullen and furious passion is controlled and governed with ease by kindness—so that only two out of several hundred had to be confined at the time of our visit, and yet into their cells could the excellent superintendent, Dr. Hansbury Smith, venture without fear of personal injury.

Columbus is beautifully situated near the center of the State, and, by the munificence of the State and the enterprise of her citizens, is fast becoming one of the most delightful places in Ohio. The Asylums, Penitentiary, State House, (and last although not least attractive) Starling Medical College, present a group of public buildings not easily surpassed. The last two are still unfinished, yet the latter will be ready for occupancy this winter.

Xenia.—This place is about two hours ride from our City, and would of itself be a pleasant retreat for our citizens in the heat of summer; but, during the past season, the Xenia Springs, three and a half miles from the place, was quite a favorite resort for many of the City—several having family cottages, very neat and inviting; and the public hotel is a pretty extensive frame building. A few years, with continued improvement, will make these springs a lovely place; and, we judge, they will be thronged every summer.

The town, with the fine country around, supports some two or three dentists —for the past year, two offices have been kept open—supply a business to some extent for four operators; and, we are glad to learn, they possess pretty generally the confidence of the public. Dr. J. Taft is a graduate of the Ohio College of Dental Surgery, and we feel assured will ever strive to place the profession on its proper footing in any community he may practice in. We have seen some operations from his hands, creditable to any operator.

Dr. Hamill, also a graduate of the Ohio College, is also of this place, and a student of Dr. Taft. The latter gentleman's method of inserting pivot teeth, although not really new, yet in his hands is made a very finished operation. The root is prepared as usual by drilling, but instead of receiving a wooden pivot, it first receives a gold tube. This is fastened sometimes by being screwed in—but oftener, we believe, by a firmly compacted gold filling around it, and which, if the root is decayed, fills it up like a plug and arrests the decay. The pivot is fastened to the tooth by solder—the tooth is heated until the solder flows into the cavity, the pivot being held to its proper place. The tooth should be gradually heated, first heating the cutting edge of the tooth. There should be a little borax in the pivot hole of the tooth. But we refer the reader to an article of the Doctor's, in the third volume of the Register. We are rather inclined to think that many in the Profession have too much discarded this operation, often extracting roots which would be serviceable for years to retain pivot teeth, if properly prepared. Let us illustrate a case: Mrs. B—— calls on Dr. A——, having lost the first two bicuspide superior—she has also two of the incisors broken off, the roots sufficiently firm to retain teeth, say five years, under proper treatment; but Dr. A—— advises the extraction of these roots and the insertion of four teeth instead of two. He is doubling the size of his operation and more than doubling the *amount* of his bill. But is he doing his patient justice? True, it may be gratifying to Mrs. B—— to have the side vacancies also filled. But is not this vacancy of service in the preservation of her other teeth? We think in nine cases out of ten it is—and as often would the clasps injure more or less, the teeth to which the artificial ones are attached.

We meet with cases frequently of this kind, and we are satisfied that the loss of a bicuspid on either side is generally of service, provided the other teeth are good, and the vacancy should not be filled up merely for ornament. But we have digressed, and will return to our Xenia friends. Drs. Payne and Stipp are also located here, and we had the pleasure of looking at some of their work, which was neatly executed. Xenia is almost the first field of our dental labor, having visited the place in the fall of 1827, and spent several weeks doing what we could professionally for the public, and Dr. Taft informed us that a certain lady still wears a pivot tooth or two we then inserted. We hope again to see our brethren of Xenia, and that they may long enjoy professional prosperity.

